# Methamphetamine-Associated Corneal Ulcer: Case Report

**DOI:** 10.3390/reports8030147

**Published:** 2025-08-17

**Authors:** Amy Conner, Brian K. Foutch

**Affiliations:** 1 Focal Point Vision, 4775 Hamilton Wolfe, San Antonio, TX 78229, USA; 2 Rosenberg School of Optometry, University of the Incarnate Word, 9725 Datapoint Drive, San Antonio, TX 78229, USA; foutch@uiwtx.edu

**Keywords:** case report, methamphetamines, corneal ulcer, amniotic membrane, illicit drug use, non-sterile ulcer, sterile ulcer

## Abstract

**Background and Clinical Significance:** This case report highlights the rare but potentially sight-threatening presentation of corneal ulcers associated with methamphetamine abuse. Identifying the signs of illicit drug use is critical, as ocular complications may be overlooked without proper social history or lab confirmation. **Case Presentation:** A 48-year-old Hispanic male presented with progressive bilateral vision loss over four weeks, describing his condition as “blind vision.” Two weeks earlier, he had visited the emergency room after a fall caused by impaired vision and was prescribed insulin, metronidazole, and fluoroquinolone drops. At ophthalmology follow-up, visual acuity was 20/400 OD and 20/800 OS. Examination revealed bilateral stromal corneal ulcers with infiltrates. Notable systemic signs—pockmarks, poor dentition, thin body habitus, and jittery behavior—raised suspicion for methamphetamine use. He was treated with bandage contact lenses, dehydrated amniotic membranes, and a steroid-antibiotic combination drop. **Conclusions:** This case underscores the importance of recognizing physical signs of methamphetamine abuse, even in the absence of disclosed history. Emergency room laboratory testing confirmed methamphetamine use. Awareness of drug-induced ocular effects allows for appropriate patient education, timely intervention, and referral to addiction services. Patients should be warned that continued drug use may result in irreversible vision loss.

## 1. Introduction and Clinical Significance

A corneal ulcer is defined as “a lesion caused by superficial loss of tissue, usually with inflammation” [1]. Most ulcers do not arise in otherwise healthy eyes; rather, there is typically an underlying cause. Ulcers can be classified as either sterile or non-sterile. Sterile ulcers are inflammatory but non-infectious in origin—arising from hypersensitivity reactions, neurotrophic conditions, exposure keratopathy, trauma, or autoimmune disorders. In contrast, non-sterile ulcers result from microbial contamination by bacterial, viral, fungal, or protozoan pathogens, with bacteria being the most common culprits [1].

Some ulcers begin as sterile due to surface compromise and later become non-sterile when opportunistic microbes infiltrate. Differentiating between sterile and infectious ulcers is critical, as it guides treatment—anti-infective agents for microbial causes, and corticosteroids or supportive therapy for sterile inflammatory cases [1].

Drug abuse, particularly methamphetamine use, is an increasingly recognized cause of both sterile and non-sterile corneal ulcers [2]. Methamphetamine’s sympathomimetic effects suppress blinking and induce lagophthalmos, leading to exposure keratopathy. Additionally, methamphetamine is often cut with caustic agents such as alkali chemicals, caffeine, talc, or anesthetic agents such as lidocaine or other compounds belonging to the amide or ester classes of local anesthetics, typically characterized by the suffix ‘-caine’, which can result in chemical burns and toxic epithelial damage [3]. The neurotrophic effects of methamphetamine are also significant. Corneal nerves express dopamine receptors, and meth-induced dopamine surges can cause corneal neurotoxicity. This results in impaired sensation and painless progression of corneal ulcers—commonly referred to as neurotrophic keratitis [2]. In such cases, ulcers may deepen into the stroma without pain, delaying presentation and worsening outcomes. 

Exposure keratopathy remains the most common ocular manifestation of methamphetamine use, regardless of the drug’s route of administration [4]. Furthermore, use of methamphetamine frequently leads to behaviors such as excessive eye rubbing, which can worsen epithelial injury and introduce infectious agents. Inhaled or smoked methamphetamine increases the risk of direct ocular exposure to chemical contaminants. 

Methamphetamine’s popularity is linked to its low cost, high potency, and long duration of effect, with a high lasting up to 24 h—far longer than that of cocaine, its more expensive stimulant counterpart [3]. Originally developed for military use during World War II to improve endurance and alertness [5], methamphetamine has become accessible through home synthesis using over-the-counter precursors like pseudoephedrine [3]. Data from the 2018 National Survey on Drug Use and Health reported that 53.2 million Americans aged 12 and older had used illicit drugs, including marijuana, heroin, prescription stimulants, and methamphetamines [6]. Methamphetamine Use Disorder—defined by recurrent use resulting in health, legal, and social problems—was diagnosed in 1.1 million people in that year alone.

Therefore, clinicians at all care levels must be vigilant for ocular signs suggestive of methamphetamine abuse—particularly corneal ulcers in young or otherwise healthy individuals with no obvious cause. Patients often withhold accurate histories, so recognition of behavioral clues (agitation, poor hygiene, “meth mouth,” pock-marked skin, dilated pupils) and ocular signs (neurotrophic ulcers, exposure keratopathy, chemical burns) is critical [2]. With methamphetamine use on the rise again in the United States, eye care professionals should include it in the differential diagnosis for unusual or non-healing corneal ulcers. Here we report our experience with a corneal ulcer in a methamphetamine user. We present a case report and discussion of rare, but sight-threatening, corneal ulcers associated with methamphetamine abuse.

## 2. Case Presentation

### 2.1. Initial Examination: Day One

#### 2.1.1. Clinical History and Objective Examination 

A 48-year-old Hispanic male presented to the ophthalmology clinic with a chief complaint of subjectively reported bilateral complete vision loss. The patient reported acute, bilateral onset four weeks prior, noting progressive deterioration since. He described current vision as limited to light perception and vague facial outlines. Visual decline was symmetric with no reported flashes, floaters, trauma, photophobia, discharge, or prior ocular disease. He denied systemic illness, medication use, or substance use, although he was an unreliable historian with minimal medical, family, or social history provided.

Two weeks prior, the patient visited an emergency room after falling into an end table, reportedly due to poor vision. He struck his forehead but denied ocular trauma. He recalled brief left eye pain post-injury and vague use of pain medications and eye drops, but details were unclear.

On exam, uncorrected visual acuity was 20/400 OD and 20/800 OS with no improvement on pinhole. Extraocular motility was full OU. Pupils and confrontation fields could not be reliably assessed. The patient appeared underweight, disoriented, anxious, and irritable, with visible skin lesions on the forearms and shins and poor dentition.

**Figure 1:**
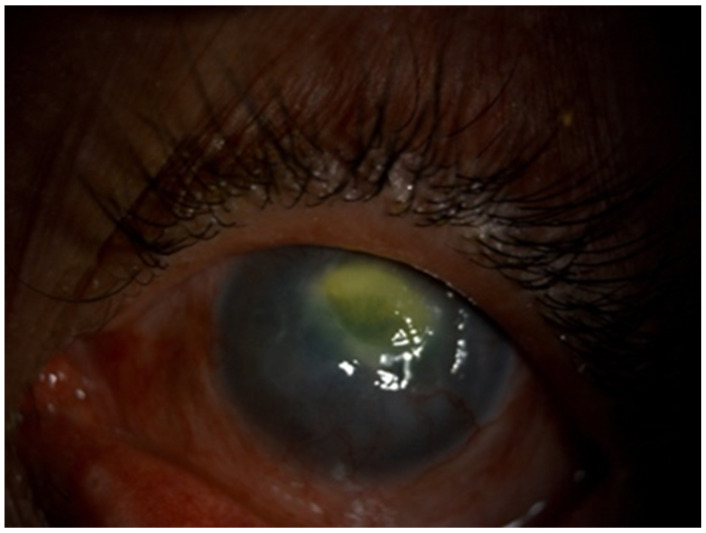
Anterior segment photograph of left eye. Note the large corneal ulcer. Right eye image was not obtainable.

Slit lamp examination revealed bilateral central corneal ulcers with posterior stromal involvement, but intact Descemet’s membranes. The left ulcer measured 4 × 3 mm with associated stromal edema (see Figure 1); the right ulcer measured 1 mm in diameter. Anterior chambers demonstrated 1+ cells and trace flare OU. No discharge was noted in either eye. Rose Bengal staining was negative OU. Intraocular pressure (IOP) was measured at 8 mmHg OD and 9 mmHg OS via rebound tonometry (iCare, Icare USA, Inc., Raleigh, NC, USA). Posterior segments could not be visualized due to corneal edema; however, B-scan ultrasonography showed flat retinas and no posterior segment inflammation OU. The patient declined corneal cultures due to cost and lack of insurance coverage.

#### 2.1.2. Differential Diagnoses 

**Bacterial microbial keratitis (non-sterile component):** The leading initial differential diagnosis was bacterial keratitis, supported by the presence of corneal infiltrates and underlying stromal edema. Typical bacterial infections are often associated with ropey, white, suppurative, or mucopurulent discharge. Although no discharge was noted in this case, the clinical suspicion remained high. It is possible that methamphetamine use led to epithelial breakdown—either through mechanical trauma (e.g., eye rubbing) or neurochemical disruption (via dopamine-related mechanisms)—permitting opportunistic pathogens to penetrate the corneal surface. Common additional signs of bacterial keratitis, such as hypopyon, pronounced anterior chamber reaction, or endothelial plaques, were absent but do not rule out this diagnosis [1].

**Drug-associated ulceration (sterile or non-sterile):** Ulceration secondary to illicit drug use—particularly methamphetamine—is the second major consideration. These ulcers may present as sterile or non-sterile depending on the route of administration and individual response. Central, painless ulcers are characteristic of drug-induced damage. This differential is highly suspected based on the patient’s physical appearance, including pock-marked skin and erratic behavior. While this etiology may be primary, it may also overlap with bacterial infection due to the compromised corneal surface [7].

**Neurotrophic Keratitis:** Neurotrophic keratitis was a strong contender, possibly coexisting with the above diagnoses. These sterile or secondary non-sterile ulcers result from reduced corneal sensation due to trigeminal nerve dysfunction. Classically, these ulcers are painless and may progress deeply without triggering protective blink or healing responses. Rolled epithelial edges are a common finding. In this case, the absence of pain despite deep stromal involvement supports this diagnosis. Later emergency department reports revealed uncontrolled Type 2 diabetes mellitus, a known risk factor for neurotrophic corneal disease [8,9].

**Fungal Keratitis:** Fungal keratitis, while less likely, remained on the differential list. These infections typically follow vegetative trauma, and the ulcers often exhibit feathery borders and slow progression. Although no such trauma was reported and the ulcer borders were not classically feathery, the patient’s unreliable history makes exclusion difficult. A suspicious superior opacity further complicates assessment. Definitive diagnosis would require corneal scraping for culture on Sabouraud agar, PCR analysis, or confocal microscopy to detect fungal elements [10].

**Herpes simplex virus (HSV) ulceration:** Herpetic keratitis is another consideration, though unlikely in this case. Classic early findings include branching dendrites stained with Fluorescein or Rose Bengal. Devitalized epithelial cells at the lesion margins stain positively with Rose Bengal. HSV typically presents unilaterally and respects the horizontal midline due to trigeminal innervation. Although HSV can lead to neurotrophic ulceration, the round, bilateral, non-dendritic, non-staining ulcers seen here do not support this diagnosis [11].

**Acanthamoeba keratitis (protozoan, non-Sterile):** Acanthamoeba keratitis, though rare, should be considered. This protozoan pathogen is typically associated with exposure to contaminated water or poor contact lens hygiene. Hallmark findings include ring-shaped infiltrates and severe pain disproportionate to clinical appearance. In this case, the patient denied contact lens wear, swimming, or water exposure and reported no pain. Therefore, this diagnosis is unlikely. Definitive testing includes confocal microscopy for trophozoites or culturing corneal scrapings on non-nutrient agar with E. coli overlays [12].

#### 2.1.3. Initial Assessment and Plan

Due to the objective findings of facial papules, thin frame, and jittery appearance, combined with a confused demeanor, amphetamine use was suspected. However, without a more complete medical history, no definitive etiology could be determined and a diagnosis of bilateral central corneal ulcers with a neurotrophic component from uncontrolled Type 2 diabetes mellitus (T2DM) was formulated with a suspicion that methamphetamine was the initial insult.

The treatment plan included placing a dehydrated amniotic membrane on the left eye first to aid in the healing process due to suspected neurotrophic etiology. A dehydrated amniotic membrane along with a plano-powered bandage contact lens was placed on the left eye. Due to insurance reasons, an amniotic membrane and bandage contact lens were not placed on the right eye the same day, and the patient was scheduled for two days later. The patient was instructed to use Maxitrol drops (Neomycin-Polymixin B Sulfate- Dexamethazone ophthalmic suspension) in both eyes six times per day. The patient was told to use the drops over the bandage contact lens OS and to not remove the bandage contact lens. A Maxitrol drop taper was not discussed as the patient was scheduled back within two days for the second eye amniotic membrane placement.

### 2.2. Records Review

Patient medical records were requested from the emergency room visit 2 weeks prior to obtain a more comprehensive medical history. They documented the chief complaint as a heavy fall following a drug overdose. Ocular findings were unremarkable for corneal ulcers. The ER note reported the following ocular exam findings: right eye (OD) with erythema and trace mucoid discharge; pupils equal, round, and reactive to light (PERRL); full range of motion in both eyes (SFROM OD/OS); no conjunctival injection in either eye (OU); sclerae anicteric OU.

Key findings from the emergency room visit included diagnoses of Type 2 diabetes mellitus (T2DM), methamphetamine use, bacterial conjunctivitis, and recurrent bacterial sepsis. Relevant laboratory and radiology results are summarized as follows:

1. **Type 2 diabetes mellitus**: Uncontrolled, with a reported fasting blood glucose level of 311 mg/dL.
2. **BUN/Creatinine ratio:** Elevated.
3. **Bilirubin**: Elevated.
4. **Electrolyte imbalance**: High chloride and low potassium.
5. **Complete blood count abnormalities**:
  - Elevated white blood cell (esp., high neutrophils but low lymphocytes) and platelet counts;
  - Elevated erythrocyte sedimentation rate;
  - Decreased red blood cell count, hemoglobin, and hematocrit.
6. **Toxicology screening**:
  - Positive for amphetamines;
  - Negative for barbiturates, benzodiazepines, marijuana, cocaine, opiates, and alcohol (EtOH).
7. **Psychiatric and substance use screening**: Positive for depression, bipolar disorder, methamphetamine use disorder, and tobacco use.
8. **CT scan**: Performed to rule out intracranial hemorrhage due to the fall; results were within normal limits.

The final diagnoses at the ER visit were recurrent *Clostridium difficile* sepsis and bacterial conjunctivitis. The patient was prescribed insulin (120 mg twice daily), metronidazole (500 mg orally three times a day for seven days), fluoroquinolone (Vigamox) eye drops (right eye only; four times daily). The emergency department recommended follow-up care with an ophthalmology clinic, primary care physician, and endocrinologist.

### 2.3. Second Examination: Day 5

The patient presented to the ophthalmology clinic three days after the scheduled appointment as a walk-in. The reason for the visit was to place an amniotic membrane plus a bandage contact lens on the right eye. Patient reported using the drops prescribed “when remembers.” Entering distance visual acuities were measured at 20/400 OD and OS with no improvement recorded on pinhole. One drop of proparacaine hydrochloride 0.5% ophthalmic solution was placed in the right eye. The previously placed amniotic membrane was noted as still in place and dissolving OS. In office treatment at this visit was placement of a dehydrated amniotic membrane OD with a plano-powered bandage contact lens in the right eye. The patient was instructed to continue Maxitrol OU six times a day for one week, then taper to QID for two weeks, then BID for two weeks, then stop. A follow up appointment was made for one week to remove bandage contact lenses for both eyes and re-check ulcers for healing. The taper in drop use was further discussed to ensure increased compliance.

### 2.4. Third Examination: Day 35

After multiple contact attempts to contact the patient for needed one-week follow-up to remove the bandage contact lenses, our patient presented to the clinic a month later as a walk-in appointment. He again reported using the drops only “when remembers.” Entering distance visual acuities were taken as 20/80+ OD and 20/100 OS. Pinhole improved visual acuities to 20/40+ OD and 20/60 OS. Tonometry with iCare was taken as 10mmHg OD and OS over the bandage contact lenses. Slit lamp evaluation with fluorescein staining indicated fully resolved ulcers OD and OS. However, residual grade 1+ corneal edema remained in both eyes and was established as the etiology of degraded vision. Poor view of pupil response and fundus remained due to corneal edema. No further treatment was established at this time, and he was instructed to return in two weeks for corneal evaluation and baseline dilated ocular exam to rule out diabetic retinopathy.

The patient was lost to follow-up after this visit.

## 3. Discussion

The complex interplay of methamphetamine’s pharmacologic, chemical, and behavioral effects creates a uniquely hostile environment for the ocular surface. Methamphetamine use can impair corneal integrity through both direct tissue damage and secondary microbial invasion. Importantly, the early manifestations may be subtle or atypical, particularly in patients who are neurologically altered or reluctant to disclose drug use [1,2].

A key contributor to methamphetamine-associated corneal ulceration is its disruption of normal blink physiology. As a potent central nervous system stimulant, methamphetamine activates the sympathetic nervous system, suppressing blink reflex and contributing to incomplete lid closure, or lagophthalmos. The resulting exposure keratopathy can initiate a cascade of ocular surface compromise [4]. Even in the absence of infection, this chronic desiccation leads to epithelial breakdown and creates an entry point for opportunistic pathogens [13].

In addition to exposure-related injury, methamphetamine compounds the risk of ulceration through chemical toxicity. The drug is frequently adulterated with substances such as talc, caffeine, caustic alkalis, and topical anesthetics like lidocaine [3,14]. These agents not only delay corneal healing but also directly disrupt epithelial cell adhesion, increasing vulnerability to mechanical and microbial insult. In cases where methamphetamine is smoked or snorted, ocular exposure to airborne contaminants further elevates the risk of epithelial compromise [15]. Beyond these surface-level injuries, methamphetamine exerts neurotoxic effects on corneal sensory nerves. The human cornea contains dopamine receptors [16], and methamphetamine’s excessive stimulation of dopaminergic pathways may damage these nerves [17]. The resulting neurotrophic keratitis diminishes corneal sensation, allowing ulcers to progress silently and painlessly into the stroma. This can delay detection and treatment, increasing the risk for scarring, secondary infection, or even perforation [18].

Clinicians should also consider the behavioral consequences of methamphetamine use. Agitation, insomnia, and compulsive behaviors—such as persistent eye rubbing—can exacerbate mechanical trauma to an already compromised ocular surface [4]. Simultaneously, malnutrition, poor hygiene, and decreased adherence to care recommendations further complicate clinical outcomes [19]. Diagnosis in these cases is particularly challenging. Patients with Methamphetamine Use Disorder often underreport or deny drug use, and their presentation may mimic more common causes of corneal ulceration. The absence of pain in a progressing ulcer, poor response to standard therapy, or concurrent systemic signs of drug use (e.g., severe dental decay, skin lesions, mydriasis) should raise suspicion for substance-induced disease [2,4].

The current literature on methamphetamine’s ocular effects remains limited. However, growing case reports and mechanistic studies suggest its impact may extend beyond ulceration to include chronic tear film instability, corneal neuropathy, and impaired epithelial wound healing [14,20]. While more research is needed to quantify prevalence and outcomes, these patterns underscore the importance of heightened clinical suspicion.

Limitations in this case include the patient’s documented history of methamphetamine use, which may compromise the reliability of subjective historical information. Additionally, the application of a bandage contact lens in a noncompliant or unreliable patient poses a significant risk, as extended wear of soft contact lenses may exacerbate a corneal ulcer. Furthermore, in the absence of corneal scraping and microbial culture, the empirical use of topical antibiotics or corticosteroids could prove detrimental, particularly if the causative organism is resistant to the selected therapeutic agents or if corticosteroid use suppresses immune response in the presence of an undiagnosed infectious etiology. Likewise, Although the case was noted to have a potential neurotrophic component due to reduced corneal nerve sensitivity associated with methamphetamine use, formal corneal sensation testing was not performed to confirm a definitive neurotrophic etiology. 

Given the patient’s unreliable history of follow-up, alternative treatment approaches—such as antifungal, antibacterial, or neurotrophic therapies—were not pursued. Standard management for neurotrophic keratitis could have included agents like cenegermin (Oxervate) or extended use of scleral lenses. Standard treatment of corneal ulcers typically involves addressing the underlying infiltrate before applying an amniotic membrane or bandage contact lens. However, in select cases, early placement of an amniotic membrane may be considered due to its well-documented antibacterial and antiviral properties [21].

## 4. Conclusions

This case contributes to the emerging recognition of methamphetamine as a direct and indirect threat to corneal health. Understanding the multifactorial pathophysiology of these ulcers—ranging from exposure keratopathy and chemical injury to neurotrophic impairment and behavioral trauma—can guide more accurate diagnosis and effective treatment strategies.

Ultimately, early identification of methamphetamine-related corneal ulcers is essential. Misclassification as purely infectious or inflammatory in origin may delay appropriate intervention and result in preventable vision loss. Eye care providers—particularly in emergency, primary care, and urban clinical settings—should consider methamphetamine as a potential etiologic factor in any atypical or recalcitrant corneal ulcer, especially in younger patients with no other clear risk factors [1,4].

## Data Availability

The original contributions presented in this study are included in the article. Further inquiries can be directed to the corresponding author.

## Abbreviations

The following abbreviations are used in this manuscript:

IOP	intraocular pressure
OD/OS	oculus dexter (right eye)/oculus sinister (left eye)
OU	oculus uturque (both eyes)
T2DM	Type 2 diabetes mellitus
